# Influence of Different Aging Environments on Rheological Behavior and Structural Properties of Rubber Asphalt

**DOI:** 10.3390/ma13153376

**Published:** 2020-07-30

**Authors:** Yuhao Zhang, Hui Wei, Yinhan Dai

**Affiliations:** 1 School of Traffic & Transportation Engineering, Changsha University of Science & Technology, Changsha 410114, China; 18390975052@163.com (Y.Z.); D15673176690@163.com (Y.D.); 2 State Engineering Laboratory of Highway Maintenance Technology, Changsha University of Science & Technology, Changsha 410114, China

**Keywords:** rubber asphalt, ultraviolet aging, rheological behavior, structural properties

## Abstract

Research on asphalt aging properties mainly focuses on temperature and ultraviolet (UV) radiation, while water, acid and salt media in natural environment are often ignored. According to the aging simulation of self-made UV environment chamber, the influence of the coupling effect of different media and UV radiation on rubber asphalt properties was analyzed through physical properties, rheological properties and micro characteristics, and the structural properties of asphalt were revealed. The results indicated that different media can accelerate the aging of rubber asphalt. The penetration, creep rate and non-recoverable creep compliance of asphalt decreased, while the softening point, rutting factor, recoverable rate, fatigue factor and stiffness modulus increased. The coupling effect between acid and UV radiation had the most impact on asphalt rutting and fatigue cracking. The rubber asphalt performed better at high-temperature after aging, and it was more prone to fatigue damage and performed worse at low-temperature. Through the oxygen absorption reaction causing asphalt aging, the intensity of the absorption peaks of the carbonyl and sulfoxide groups increased and the light components of the asphalt transitioned to heavy components under UV radiation. These media intensified this process, which resulted in the gradual transformation of the asphalt colloid structure into a gel type. The carbon black contained in the waste rubber powder made the rubber asphalt exhibit better anti-aging properties.

## 1. Introduction

In the long-term use of asphalt pavement, due to the influence of environmental factors, such as UV radiation, oxidation and high-temperature, the internal structure of asphalt will change, causing pavement damage [1,2,3]. Thermal oxygen aging and UV aging are the two main forms of asphalt aging [4,5]. Thermal oxygen aging can affect the physical properties of asphalt [6,7,8]. UV aging can break the chemical bond in asphalt and recombine with oxygen, which will has a serious impact on the rheological properties of asphalt [9,10,11]. The rubber asphalt prepared by rubber powder cannot only improve the properties of asphalt, but also recycle the waste materials environmentally [12,13]. Furthermore, rubber asphalt is less expensive than styrene butadiene styrene (SBS) or epoxy resin modified asphalt [14,15]. Therefore, it is essential to reveal the aging behavior of rubber asphalt for the application of rubber asphalt.

Researchers mainly study the aging behavior of asphalt by indoor simulation aging. For example, by simulating the short-term and long-term aging of asphalt in the laboratory, Wang et al. [16] studied the effect of modifier on anti-aging properties of asphalt. Tang et al. [17] also studied the effect of sulphur on the anti-aging properties of rubber asphalt on the basis of simulating the aging of asphalt in laboratory. The above researchers used thin-film oven test (TFOT) or rolling thin-film oven test (RTFOT) to simulate short-term aging, and pressure aging vessel (PAV) test to simulate long-term aging [18,19]. However, due to the lack of UV radiation, these test conditions cannot fully simulate the actual environment of pavement [20,21,22]. Moreover, the asphalt aging conditions selected above are relatively simple, but the actual environment of asphalt is relatively complex. For example, water is a medium that accelerates the aging of asphalt under the combined effects of time, heat, oxygen and sunlight [23,24,25]. Traxler [26] listed 15 factors that cause asphalt aging and found that water accelerates asphalt aging under the combined effects of time and sunlight. In addition, water in nature is not pure water, often accompanied by other components. Acid rain, as a corrosive material, has been widely studied in the field of construction, but little research has been conducted on asphalt aging [27,28]. In addition, to remove snow and ice from pavements, melting salt is often applied, which can lead to pavement damage. Feng et al. [29] studied the influence of salt on asphalt through the basic test of asphalt, and the results showed that salt had a great influence on the low-temperature properties of asphalt, and accelerated the damage of asphalt mixture. Xiong et al. [30] studied the properties change of asphalt mixture under water salt erosion by the self-designed dynamic water-salt erosion apparatus of asphalt mixture. The results showed that salt solution would penetrate into the cracks of asphalt mixture, and the existence of water accelerated the process. Therefore, based on the above research, it is necessary to study the UV aging behavior of rubber asphalt in different environments.

Systematic studies of asphalt aging should thus consider the effects of acid and salt, which should be carried out under conditions that incorporate water. The main purpose of this paper is to grasp the aging behavior of rubber asphalt under the coupling effect of different environmental factors. Based on the short-term aging, this study used a self-made UV environment simulation chamber to carry out UV aging of base asphalt and rubber asphalt in different environments of water, acid and salt. The aging behavior and properties change of rubber asphalt under different environmental conditions were revealed by using routine index tests and rheological properties tests. The micro characteristics of rubber asphalt aging behavior was analyzed by four-component analysis, Fourier transform infrared spectra (FTIR) and scanning electron microscope (SEM), which provided help for the development of rubber asphalt.

## 2. Materials and Experimental Methods

### 2.1. Materials

Base asphalt with a penetration grade of 70 was used in the test (produced by Sinopec Maoming Branch (Maoming, China)), and its properties are shown in Table 1. The fineness of the waste rubber powder was 80 mesh. Table 2 presents the physical and chemical parameters of the waste rubber powder. The rubber powder content was 20% (by weight of asphalt) according to the results of the literature review [31,32,33]. Table 3 presents the main properties of rubber asphalt. Compared with the base asphalt, the penetration and ductility of rubber asphalt decrease, and the softening point increases. The results show that the asphalt become hard, while the plasticity and temperature sensitivity decrease. According to American Society for Testing and Materials (ASTM) test requirements, combined with our test conditions, three samples were selected for parallel testing. The asphalt used in this paper was, generally, the asphalt liquid formed after heating.

### 2.2. Simulations of Different Environmental Environments

In order to simulate different environmental conditions, water, acid and salt solution were prepared according to the method of references [34,35]. Water was prepared by using a distillation test. The concentration of the acid solution was pH = 3, ${c(\mathrm{SO}}_{4}^{2-}):{c(\mathrm{NO}}_{3}^{-})=9:1$. The chloride solution was prepared by sodium chloride crystal and distilled water to prepare 7% chloride solution. Acid solution and salt solution were mainly used to simulate the acid rain environment and the influence of snow melting salt and saline soil water on asphalt properties.

### 2.3. Simulation of Aging Process

**Figure 1:**
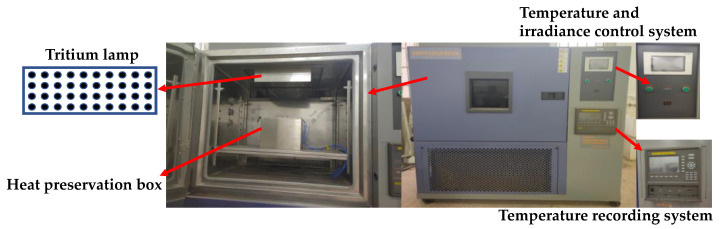
Ultraviolet (UV) environment simulation chamber and its components.

The influence of UV aging on asphalt was generated during the actual use of asphalt pavement. In order to better simulate the actual aging process, it was usually necessary to perform short-term aging treatment on the asphalt samples before UV aging [36]. According to ASTM D1754, a thin-film oven test (TFOT) was used to simulate the short-term aging process of asphalt during mixing, transportation and paving. The oven-aged rubber asphalt and base asphalt samples were placed in the self-made UV environment simulation chamber for UV radiation, after the short-term aging process. As shown in Figure 1, the external size of the device was 70 cm × 70 cm × 170 cm and the irradiation head was equal to 383 mm × 145 mm. A total of 4 rows were set with UV-LED beads, with 10 in each row and each bead was equipped with a filter to purify visible light. A quartz glass was used to concentrate light. In order to eliminate the influence of temperature on UV aging, the environmental simulation chamber was equipped with a temperature control system. During the test, the internal temperature of the chamber and the main UV wave were set to be 25 °C and 365 nm, respectively. The UV aging tests were set to four modes, namely, UV, UV and water media, UV and acid media, and UV and salt media. Due to the time-consuming nature of the UV aging test, few samples were prepared each time for each mode. For the selection of UV aging time of asphalt, most domestic and foreign scholars had adopted the aging time less than 15 days [37,38,39]. Based on the reference to the relevant literature, 7 days were chosen as the aging time to carry out the UV irradiation process. At the same time, to simulate the adverse environmental conditions of asphalt pavement, water was used to accelerate the UV aging, the environmental chamber was opened every 12 h, and 1 g of water/acid/salt was sprayed on the asphalt surface of each corresponding specimen [34]. Therefore, the aging procedure before each test was to conduct TFOT before UV aging of asphalt.

### 2.4. Routine Index Test

Using the parallel tests, penetration (25 °C) and softening point of base asphalt and rubber asphalt were measured by using ASTM D5 and ASTM D36, respectively, both before and after aging.

### 2.5. Rheological Properties Test

In accordance with ASTM D7175, the dynamic shear rheometer (DSR, Physica MCR 301, Anton Paar Instruments, Ostfildern, Germany) was used in this study to evaluate the high-temperature rutting resistance and medium-temperature fatigue of the base asphalt and rubber asphalt. A 25-mm parallel plate was used with a fixed frequency equal to 10 rad/s. Complex modulus (|G^*^|) is ratio of peak stress to the peak strain in harmonic sinusoidal oscillation, which can be used as an index to express the combined effects of viscosity and elasticity of asphalt. The phase angle (*δ*), complex shear modulus (|G^*^|) and rutting factor (|G^*^|/sin δ) were measured by using the strain variable. The medium-temperature fatigue test was conducted by using an 8-mm test plate, and the fixed test frequency and the strain were equal to 10 rad/s and 1 percent, respectively. The initial test temperature was equal to 28 °C, and the fatigue factor (|G^*^|sin δ) value of the asphalt after aging was measured every 3 °C until the fatigue factor was greater than 5 MPa.

The multiple stress creep recovery (MSCR) test is an experiment developed to resolve the problem that the original evaluation system was not applicable to modified asphalt. MSCR test can better reflect the nonlinear viscoelastic response of modified asphalt, and there is a good correlation between the non-recoverable creep compliance (*J_nr_*) and the rutting resistance of asphalt. Asphalt samples used in this test were made from asphalt after short-term aging test. The instruments and parallel plates used in the article have the same requirements as the high-temperature classification test. The specific process of MSCR was as follows: According to ASTM D7405, two stress levels of 0.1 and 3.2 kPa were selected for continuous testing. Besides, 20 cycles and 10 cycles were performed at the stress level of 0.1 kPa and 3.2 kPa, respectively, where the duration of each cycle was equal to 10 s. Each cycle was divided into a load creep phase of 1 s and an unload recovery phase of 9 s. The total duration of the test was equal to 300 s, in which the first 10 cycles at the stress level of 0.1 kPa were used in the condition of sample. The device automatically collects strain data during each creep recovery cycle. The creep recovery rate (*R*) and the non-recoverable creep compliance (*J_nr_*) of each cycle of asphalt were calculated as follows Equations (1) and (2):

(1) $$R=\frac{\gamma_{p}-\gamma_{nr}}{\gamma_{p}-\gamma_{0}}\times 100\%$$

(2) $$J_{nr}=\frac{\gamma_{nr}}{\tau }$$

where $\gamma_{p}$ is the peak strain, $\gamma_{nr}$ is the residual strain, $\gamma_{0}$ is the initial strain and τ is the creep stress. The above data can be measured by the system. Then, the system automatically calculates the average value of *R* and the average value of *J_nr_* in 10 cycles under the stress levels of 0.1 and 3.2 kPa respectively.

The bending beam rheometer (BBR, Cannon Company Instruments, State College, PA, USA) was used to measure the low-temperature properties of the base asphalt and rubber asphalt according to ASTM D6648 and AASHTO T313. The loading time was equal to 240 s. Load and deformation data were collected at 8 s, 15 s, 30 s, 60 s, 120 s and 240 s. The creep stiffness modulus (*S*) and creep rate (*m*-value) was measured for the rubber asphalt and base asphalt beams (127 mm × 6.35 mm × 12.7 mm) at −6 °C, −12 °C, −18 °C and −24 °C.

### 2.6. Aging Structural Characteristics Analysis

According to ASTM D4124, the asphalt was isolated by using organic solvents to determine the content of four components in the base asphalt and rubber asphalt. The sample was filtered with *n*-heptane solution to obtain soluble fraction. The insoluble fraction was refluxed with *n*-heptane solution and toluene solution to obtain asphaltene. Then the *n*-heptane solution containing soluble fraction was adsorbed on the pipette containing activated alumina and washed with *n*-heptane solution. After continuous washing with different solvents (toluene, toluene and ethanol, and ethanol), saturates, aromatics and resins were obtained respectively. The structural components of the asphalt were qualitatively and quantitatively analyzed before and after aging using a Fourier transform infrared spectrometer (FTIR, Bruker Tensor 27, Karlsruhe, Germany). The test range was from 4000 cm^−1^ to 400 cm^−1^, the wave number accuracy was equal to 0.01 cm^−1^/2000 cm^−1^, and the absorption accuracy was equal to 0.01 percent. During the test, 0.1 g asphalt sample was weighed and dissolved in 2 mL carbon tetrachloride. Then, the solution is dripped onto the potassium bromide window of the existing carbon tetrachloride solution, it was briefly dried, and then a film sample was formed for detection. The functional group index of asphalt was calculated according to the following Equations (3) and (4), so as to make a comparative analysis of the aging condition:

(3) $$I_{S=O}=\frac{A_{1031}}{A_{1375}+A_{1460}}$$

(4) $$I_{C=O}=\frac{A_{1697}}{A_{1375}+A_{1460}}$$

In the above formula, *A*_1031_ is the absorption peak area of sulfoxide group, *A*_1697_ is the absorption peak area of carbonyl group and *A*_1375_ and *A*_1460_ are the absorption peak areas of methyl group and subunit group, respectively.

At the same time, the aging samples of the base asphalt and rubber asphalt under the different environmental conditions were scanned using scanning electron microscopy (SEM, S-3000N, Hitachi Limited, Tokyo, Japan) to observe the surface aging cracking characteristics of the samples. The SEM maximum voltage was equal to 30 kV, magnification was equal to 0–10,000, and the maximum resolution was about 5 nm [35].

## 3. Results and Discussion

### 3.1. Physical Properties of Asphalt

The penetration and softening point are the most basic properties indices of asphalt. The penetration degree reflects the consistency viscosity of asphalt. The smaller the penetration degree is, the greater the consistency of asphalt. The softening point of asphalt reflects the high-temperature viscosity of asphalt. A higher softening point of asphalt indicates greater viscosity and better temperature stability. Table 4 shows the changes in penetration and softening points, respectively, related to base asphalt and rubber asphalt after UV aging in different media. The results prove that, compared with the penetration of asphalt after short-term aging, the penetration of the base asphalt and rubber asphalt decreases after UV aging. The softening point of asphalt is mainly affected by its asphaltene content. Photocatalysis causes a change in the molecular structure of the asphalt. A large number of C-H, C-C, and C=C bonds are present in the molecular structure of asphalt, and under the action of UV rays, the molecules absorb energy and cause these chemical bonds to break, which in turn cause the transfer of the light components in the asphalt to become heavy components. Aging makes the asphalt brittle and hard, resulting in a decrease in the penetration level and an increase in the softening point. Rubber asphalt contains a large amount of waste rubber powder. During the UV aging process, waste rubber powder has different degrees of cracking that produce free radicals. The main chain breaks up to form a new cross-linking structure, and the rubber powder interacts with the asphalt components to enter into asphalt colloid structure. This process causes the asphalt to harden, which plays an important role in improving the asphalt anti-aging properties. This process shows that the penetration and the softening point is better. Among the four UV aging modes, the changes in the penetration and softening points of the asphalt after aging in acid and salt media are greater than those in the water medium [40]. This phenomenon shows that water accelerates the UV aging of asphalt and the cracking of rubber powder, and acid or salt components further accelerate the aging and cracking process.

### 3.2. Rheological Behavior Analysis

#### 3.2.1. High-Temperature Properties

**Figure 2:**
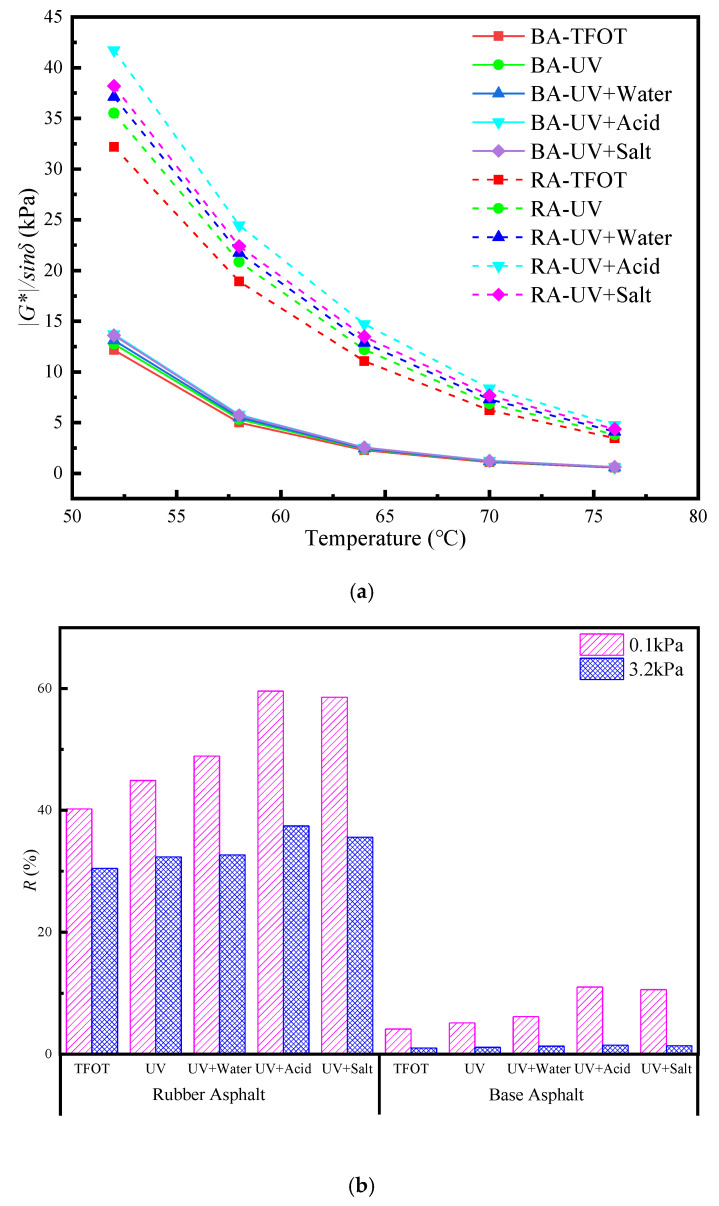

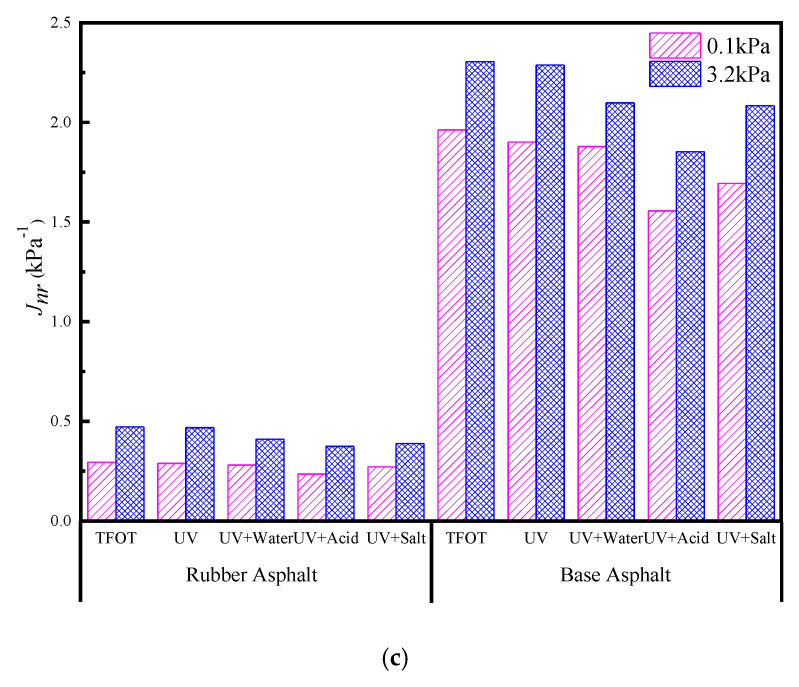
(**a**) Asphalt rutting factor after UV aging under different environmental conditions. Note: BA is base asphalt and RA is rubber asphalt; (**b**) *R* after UV aging under different environmental conditions (60 °C); (**c**) *J_nr_* after UV aging under different environmental conditions (60 °C).

The rutting factor (|G^*^|/sin δ) shows the permanent deformation resistance ability of the asphalt, where the larger value indicates a better rutting resistance of the asphalt. The non-recoverable creep compliance (*J_nr_*) refers to the ratio of residual strain to the stress of the asphalt material after creep and recovery cycles. A smaller value indicates a stronger ability for asphalt to resist permanent deformation. The average percent recovery (*R*) refers to the recovery ability of asphalt after creep. A higher *R* value indicates a stronger high-temperature resistance to deformation [31]. Figure 2 shows the variation of rutting factor, *R*, *J_nr_* after short-term aging and UV aging of base asphalt and rubber asphalt based on DSR and MSCR. After analyzing the results from Figure 2, it is possible to conclude that the rutting factor value and *R* of the asphalt after UV aging are higher than those of the short-term aging asphalt, and *J_nr_* is lower than that of the short-term aging asphalt. The rutting factor, *J_nr_* and *R* of UV-aged base asphalt and rubber asphalt are similar in different media. These results show that the temperature sensitivity decreases, the complex modulus increases, the rutting factor increases, the *R* increases, the *J_nr_* decreases and the high-temperature properties of the asphalt improves. In an acid medium, the rutting factor value of asphalt is the highest, *R* is the largest, *J_nr_* is the smallest, and rutting resistance is the best. Under the action of acid, acidic substances, such as carboxylic acids and phenols in the asphalt, dissolve and ionize in the acid solution. The ions in the solution react with components in the asphalt, resulting in a decrease of asphalt binder and acceleration of the aging of the asphalt. Under the action of salt, the salt penetrates the cracks, adheres to the asphalt surface and reduces its bonding strength, which negatively affects the asphalt properties in the road when it is subjected to UV radiation. However, the rubber powder polymer in rubber asphalt will move in segments, whereby some polymers with a lower molecular weight will flow into the asphalt and the light components of the asphalt will diffuse into the rubber powder, which promotes the expansion of the rubber powder. Such expansion, or swelling, makes the heat of the rubber powder relatively more uniform and less prone to carbonization, which improves the anti-aging ability of the asphalt [41]. The rubber powder contains a small amount of carbon black. Carbon black is an excellent light-shielding agent and thermal oxygen stabilizer. Due to the degradation of rubber powder and the release of carbon black, the thermal oxygen aging and UV aging properties of asphalt can be significantly improved, so the high-temperature deformation resistance and elastic recovery ability of asphalt improve [42].

#### 3.2.2. Moderate-Temperature Properties

**Figure 3:**
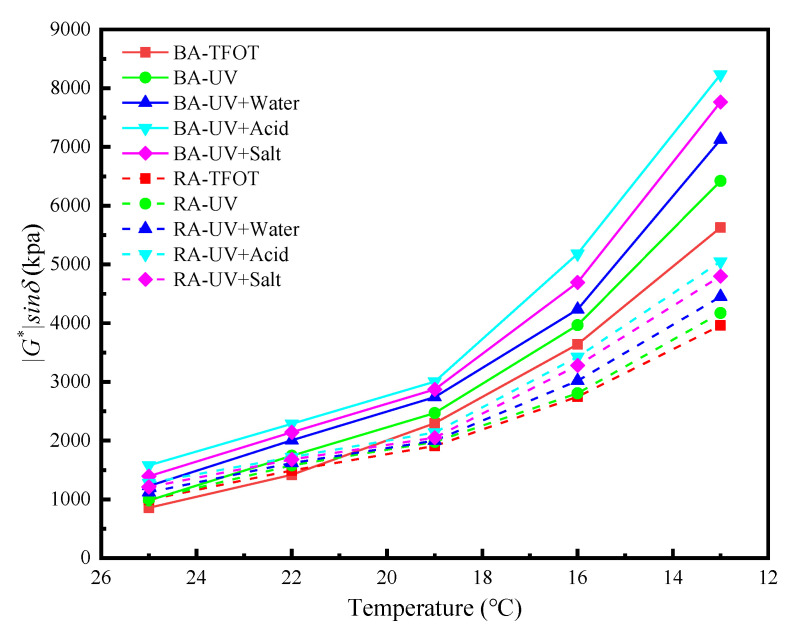
Asphalt fatigue factor after UV aging under different environmental conditions.

The fatigue crack resistance of the asphalt is represented by the fatigue factor (***|***G^*^|sin δ). The greater the fatigue factor value at the same temperature, the more likely fatigue damage of the asphalt occurs. Figure 3 presents the short-term aged base asphalt and rubber asphalt test specimens in terms of the high-temperature DSR test results for UV aging. The results from Figure 3 prove that the fatigue factor value gradually increases with the decrease of temperature for both base asphalt and rubber asphalt under the different conditions. The fatigue factor value of both base and rubber asphalt increases after UV aging and is the highest in the acid medium. The reason for this outcome may be that asphalt dissolves some of its liquid saturates and aromatics in water, which promotes the aging of the asphalt and leads to an increase in the fatigue factor. In the acid or salt medium, the presence of acid or salt accelerates this process and the aging of the asphalt, as well as weakening the asphalt fatigue cracking resistance and diminishing road properties. For the rubber asphalt, the oil in the asphalt reacts with the rubber to form a gel, which makes the rubber asphalt form a relatively stable solid-liquid two-phase structure. The rubber is connected to the liquid base asphalt through the gel so that the asphalt gains rubber resistance. Therefore, the fatigue factor value of the rubber asphalt is lower than that one of the base asphalt, and consequently, the anti-aging properties of the rubber asphalt are better than that one of the base asphalt [43].

#### 3.2.3. Low-Temperature Properties

**Figure 4:**
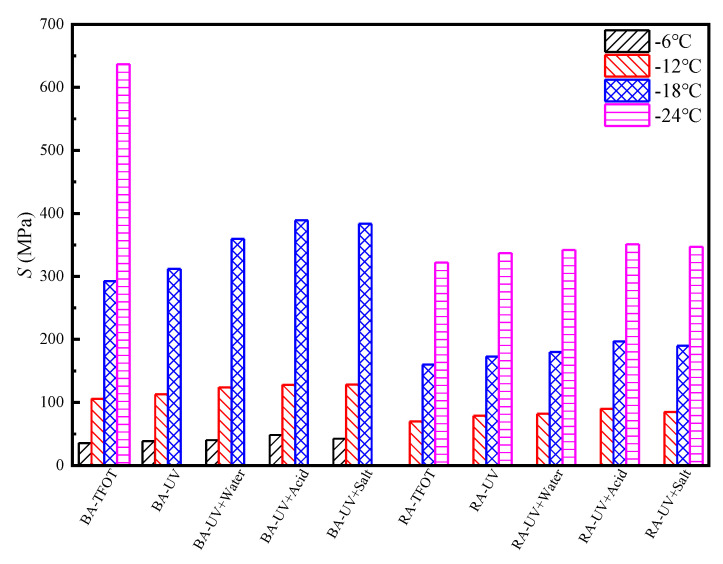
The stiffness modulus (*S*) of asphalt after UV aging under different environmental conditions.

**Figure 5:**
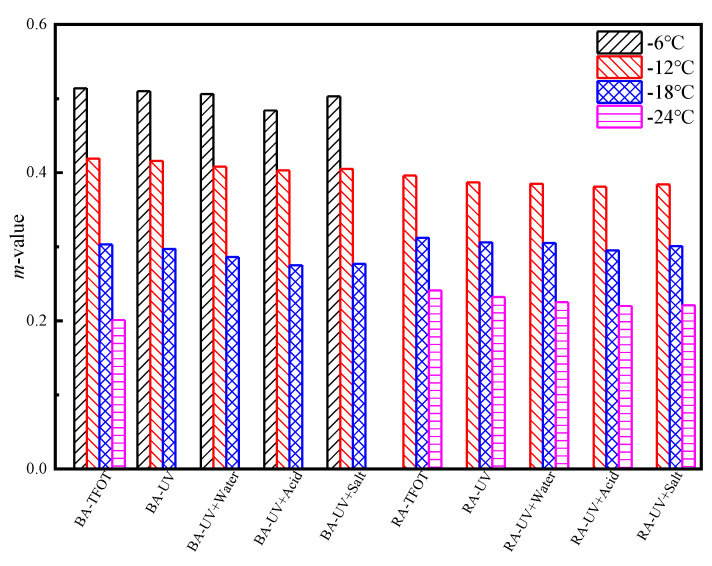
The creep rate (*m*-value) of asphalt after UV aging under different environmental conditions.

The creep stiffness modulus and *m*-value reflect the low-temperature properties of asphalt. Figure 4 and Figure 5 present the short-term aging test results for the base asphalt and rubber asphalt, as well as the low-temperature BBR test results for UV aging. The results from Figure 4 and Figure 5 prove that when the temperature decreases, the stiffness modulus increases and *m*-value decreases, which indicates that the deformation resistance of asphalt deteriorates at low-temperature and is prone to cracking. Rubber asphalt has good low-temperature properties, and its stiffness modulus is very small at −6 °C. Therefore, rubber asphalt should be tested at −12 °C. The stiffness modulus of base asphalt and rubber asphalt increases while the *m*-value decreases after UV radiation in water medium, and this phenomenon is aggravated in the acid or salt medium. This phenomenon occurs because the saturated and aromatic components of the asphalt are soft and play a plastic role, whereas the resin and asphaltene are hard components and act as a thickener. The ratio between soft components and hard components corresponds well with the rheological indices. In addition, during the process of UV radiation, various chromophores present in the pitch molecules absorb energy from the UV rays and this phenomenon produces an excited state from the ground state, causing the chemical bonds to break. The pitch changes from solid to gel, and from colloid to solid, meaning that the pitch becomes brittle and hard, which causes its stiffness modulus increases and *m*-value decreases [44]. Under the continuous action of high-temperature, sulfur and other chemical substances in the rubber particles enter and modify the asphalt, which improves the low-temperature properties and high-temperature fluidity of the rubber asphalt. Therefore, the deformation resistance of rubber asphalt is better than that one of the base asphalt. In an acid or salt solution, the ionic phase of the solution reacts with the asphalt components or adheres to the surface layer to accelerate the aging of the asphalt, causing the asphaltene content to increase and the aromatic content to decrease; thus, the proportional relationship between the soft and hard components changes significantly. The asphalt becomes brittle and hard, the stiffness modulus increases, the *m*-value decreases, and the resistance to deformation is poor.

### 3.3. Four-Component Analysis

**Figure 6:**
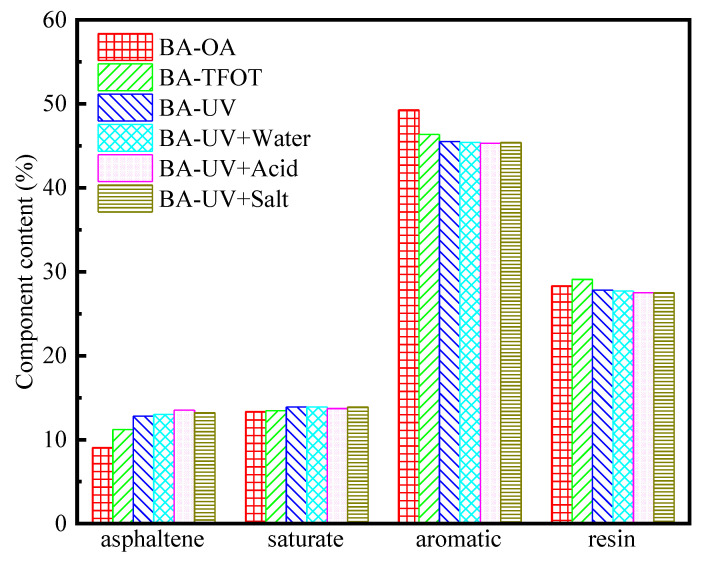
Four components of base asphalt after UV aging under different environmental conditions. Note: BA-OA is base asphalt-original asphalt.

**Figure 7:**
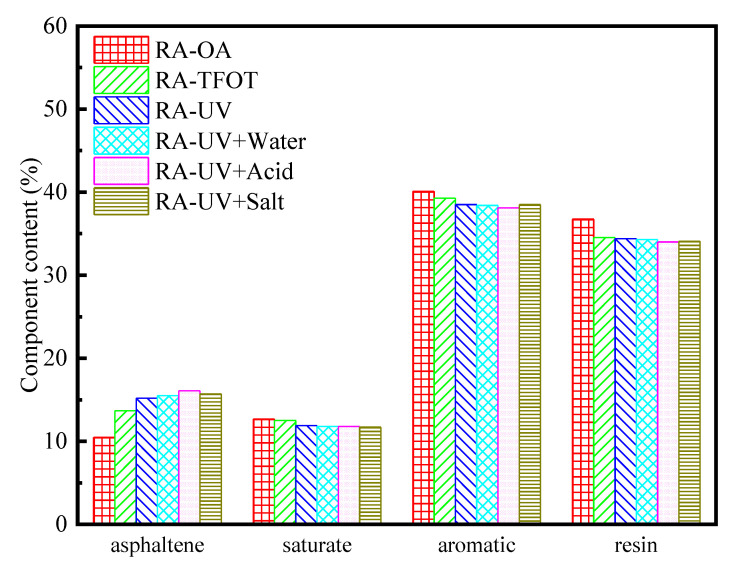
Four components of rubber asphalt after UV aging under different environmental conditions. Note: RA-OA is rubber asphalt-original asphalt.

Asphalt is a mixture consisting of a variety of compounds with a complex structure. The asphalt is usually divided into four components with similar properties according to the solubility of asphalt in different solvents. Figure 6 and Figure 7 show the aging results of base asphalt and rubber asphalt, respectively, in terms of the four-component analysis of UV aging. After analyzing the results from those figures, it is possible to ascertain that, on the whole, aging causes light components to migrate like heavy components. The molecular weight of the saturated component is small, and its properties stable, so its content changes little before and after aging. In the base asphalt, the content of saturation slightly increases after aging, while in the case of rubber asphalt, slightly decreases. This observation can be explained by the fact that the change of saturated content mainly depends on the volatilization and decomposition of macromolecular substances in different aging modes. Rubber asphalt can inhibit the decomposition reaction of asphalt in the aging process to some extent. Most of the aromatic components are transferred to the asphaltenes and resins during the aging process, resulting in a decrease in the overall content of the aromatic components. Resin is very sensitive to UV radiation and is easily stimulated by UV radiation to produce asphaltene. As an intermediate product that results from the transition from an aromatic component to asphaltene, the resin is affected by the rate of the transition from the aromatic component to resin and from the resin to asphaltene. Asphaltene is a type of heavy component with a high molecular weight. Some substances with low molecular weight volatilize and some substances with heavy components polymerize during the aging process, which leads to a large increase in the content of the asphaltene.

Under UV radiation, the change trends of the different asphalt components are similar for the water, acid and salt conditions, which promotes the aging of the asphalt to a certain extent. The UV aging of asphalt mainly occurs on the surface. During the aging process of asphalt, more free radicals are generated, and the existence of water accelerates the movement of free radicals, thereby promoting the aging of asphalt [45]. In a salt solution, the light components, such as the carboxylic acids and phenols in the asphalt, oxidize when exposed to water, which causes the salt medium to accelerate the oxidation of the asphalt in water. The aging of asphalt is most significant in the acid solution, which may be due to the dissolution and ionization of carboxylic acids and phenols in the acid solution. In the acid solution, the ion reacts with the group in the asphalt to produce acid corrosion and further promotes the UV aging of asphalt. Rubber asphalt is a type of waste tire rubber powder added to the base asphalt after processing. The molecular weight of the waste tire rubber powder is large, so the four-component analysis shows that the asphaltene and resin content of rubber asphalt is high. The resin and aromatic content in asphalt are basically the same under different aging conditions, while the content of the same component in base asphalt varies in different aging conditions. Therefore, rubber asphalt can retard or inhibit the aging of asphalt to a certain extent, and its anti-aging properties are better than those of the base asphalt [46].

### 3.4. Infrared Spectra Analysis

**Figure 8:**
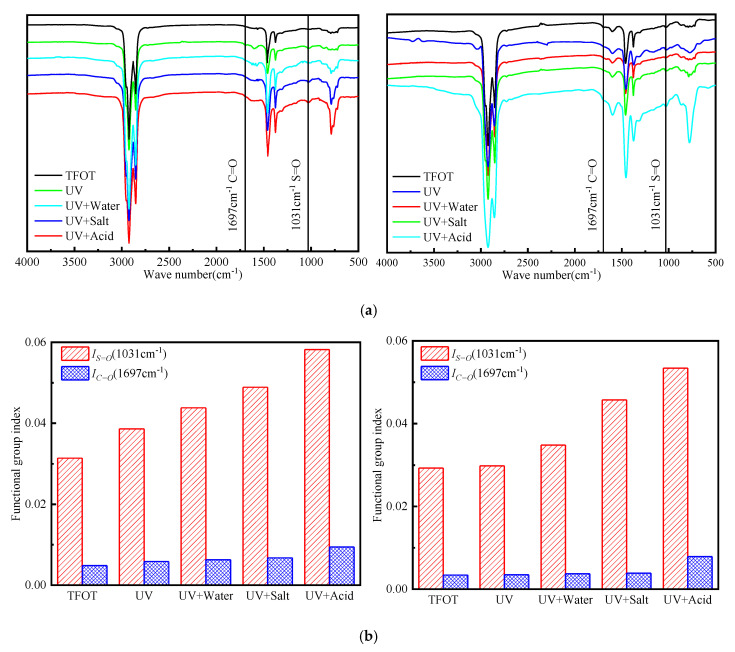
(**a**) Infrared spectra of base asphalt (left) and rubber asphalt (right) under different environmental conditions. (**b**) Functional group index of base asphalt (left) and rubber asphalt (right) under different environmental conditions.

In the analysis of the infrared spectra, the functional group region is generally identified by its characteristic stretching vibration. The presence of the functional group and the combination of other groups are further confirmed according to the absorption. Some relevant research results show that the carboxyl group and sulfoxide group can be used to characterize asphalt aging [37,47]. Both the carbonyl group and sulfoxide group are products of a chemical reaction in which the molecular composition of the asphalt is oxygen-absorbing. The greater the change in the carbonyl and sulfoxide groups, the more severe the oxygen aging of the asphalt [48]. Figure 8 shows the infrared spectra and functional group index of carbonyl and sulfoxide groups of base and rubber asphalt in different environments. With the analysis of the results in Figure 8, it is possible to conclude that the aging degree of asphalt can be judged by observing the trend of infrared spectra and comparing the change of functional group index. The wave number at 1031 cm^−1^ is the characteristic peak of the sulfoxide group. Compared with TFOT, the increase of functional group index of base asphalt after UV aging is higher than that one of rubber asphalt, which indicates that the aging degree is more serious. In different environments, the sulfoxide functional group index of asphalt does not significantly increase compared with TFOT, and the degree of UV with acid aging is more serious, followed by UV with salt or UV with water, and UV is lighter. The characteristic peak of the carbonyl group is at the wave number of 1697 cm^−1^. In different UV aging environments, the trend of infrared spectra and functional group index of the carbonyl group are similar to that one of the sulfoxide group, which shows that UV is lighter, UV + acid is more serious, and the variation value of functional group index can be greater than that of TFOT. The carbonyl group that is produced after UV aging is not obvious. The reason for this fact is that the formation of the carbonyl group is the bond-breaking oxidative reaction of the carbon chain. This reaction will continue under the continuous influence of heat and oxygen. Under the UV radiation, high-energy photons can stimulate an oxygen absorption reaction. However, because UV radiation can only radiate asphalt to a limited depth below the pavement surface, the photo-oxidative reaction and rate of increase of the carbonyl group become increasingly slower [44]. The formation of the sulfoxide group is also related to the content of the sulfur element in the asphalt. On the one hand, the sulfur element in an asphalt molecule can be easily oxidized, allowing for the aging process to nearly complete during short-term aging. Besides, aging may be caused by the short time of UV radiation, which does not cause serious UV aging of asphalt.

### 3.5. Scanning Electron Microscopy

**Figure 9:**
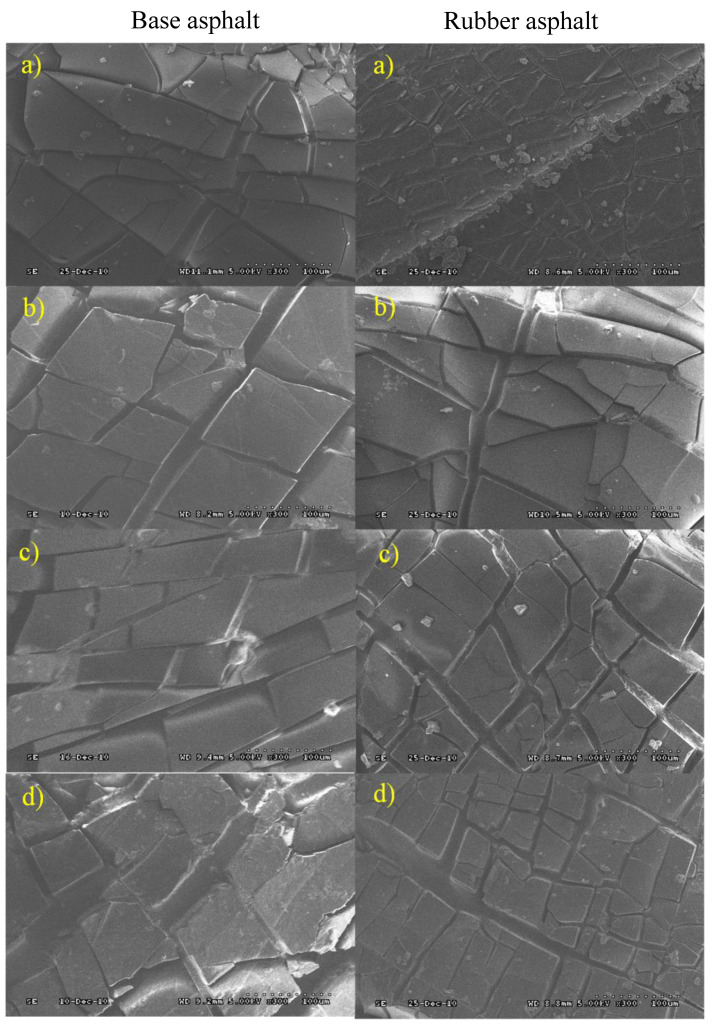
Appearance topography after UV aging of base asphalt and rubber asphalt in different environmental conditions (300 times): (**a**) UV; (**b**) UV + water; (**c**) UV + salt; (**d**) UV + acid.

The cracking of the asphalt surface can be used to characterize the degree of aging, where more obvious fractures of the surface cracks indicate a more serious degree of aging. Figure 9 presents the short-term aging results of base and rubber asphalt and the structural characteristics after UV aging. After analyzing the results from Figure 9, it is possible to state that, under UV radiation, cracks appear on the asphalt surface, as well as deeper cracks appear on the base asphalt, which are wider than in rubber asphalt. Aging is the most serious factor in the acid environment. For the base asphalt, the degree of fragmentation is aggravated in the acid environment. The cracks are deep and particles appear, whereas the cracks in the rubber asphalt are more regular and narrower. In the salt environment, the surface of the base asphalt is covered by salt and shows irregular cracking patterns and deep cracks. However, the crack distribution in the rubber asphalt is more regular, and the depth and width of the cracks significantly decrease. Asphalt is extremely sticky. Rubber powder has a strong mutual stickiness with asphalt, due to its small volume, large quantity and elasticity. This slows down or prevents the growth of cracks, further improves the rheological properties of the asphalt and improves the cracking resistance of asphalt.

## 4. Conclusions and Recommendations

In this paper, the aging behavior of rubber asphalt under UV irradiation was studied from the perspectives of macro properties, microstructure and apparent morphology in water, acid and salt environments. Considering all experimental results in this study, the following conclusions can be drawn:

1. High temperature, UV radiation and water can act as adverse environmental factors for asphalt pavement and accelerate the asphalt aging process. The aging of asphalt is also accelerated in environments with acid rain or salt solution erosion.
2. After UV aging, the physical properties of asphalt diminish, whereby the penetration level decreases, the softening point increases, and the asphalt becomes brittle and hard. The rutting factor, *R*, fatigue factor, and stiffness modulus increase, *J_nr_* and *m*-value decrease and the rutting resistance of asphalt improves, but fatigue cracking and temperature shrinkage cracking are readily apparent.
3. In different environments, the swelling/expansion of the waste rubber powder in rubber asphalt slows the rubber powder’s ability to crosslink between polymer molecules. After UV radiation, the molecules in the asphalt C-H, C-C, and C=C bonds break due to aging.
4. The light components in asphalt migrate to heavy components, and the asphalt gradually transforms from a solid/gel type to a gel type, resulting in a decrease of the low-temperature deformation ability of the asphalt. This is because the light-shielding effect of carbon black in waste rubber powder can alleviate the UV aging behavior of asphalt. Although the aging law is roughly the same for the base asphalt and rubber asphalt, the anti-aging properties of rubber asphalt are better than that those of base asphalt overall.

## Figures and Tables

**Table 1:** Main properties indices of base asphalt.

Properties Indices	Technical Requirement	Measured Value	Experiment Methods
Penetration (25 °C), 0.1 mm	60–80	62.1	ASTM D5
Penetration index value, PI	−1.5–1.0	0.6	ASTM D5
Softening point, °C	≥46	47.6	ASTM D36
Ductility (15 °C), cm	≥100	>100	ASTM D113
Wax content, %	≤2.2	1.9	ASTM D721
Density (15 °C), g/cm^3^	-	1.029	ASTM D70
Dynamic viscosity (60 °C), Pa·s	≥180	217	ASTM D2171

**Table 2:** Physical and chemical parameters of waste rubber powder.

Test Items	Measured Value	Technical Requirement
Residue, %	0.6	<10
Relative density, g/cm^3^	1.28	1.10–1.30
Moisture, %	0	<1
Metal content, %	0.005	<0.03
Fiber content, %	0.015	<1
Ash	7.2	≤8
Acetone extract	7.3	≤16
Carbon black content	29.1	≥28
Rubber hydrocarbon content, %	59	≥48

**Table 3:** Main properties indices of rubber asphalt.

Properties Indices	Technical Requirement	Measured Value	Test Methods
Penetration (25 °C), 0.1 mm	50–70	57.9	ASTM D5
Softening Point, °C	>58	64.2	ASTM D36
Ductility (5 °C), cm	>10	10.4	ASTM D113
Wax content, %	≤2.2	2	ASTM D721
Density (15 °C), g/cm^3^	-	1.058	ASTM D70

**Table 4:** Physical property indices of base asphalt and rubber asphalt.

Asphalt	Property Index	Original	TFOT	UV	UV + Water	UV + Acid	UV + Salt
Base Asphalt	Penetration (0.1 mm)	62.1	44.1	36	33.5	30.3	31.7
	Softening Point (°C)	47.6	53.2	55	55.5	58	56.5
Rubber Asphalt	Penetration (0.1 mm)	57.9	40	30.1	28.6	24	24.5
	Softening Point (°C)	64.2	69	71.5	71.9	75.3	73.6
